# When Better Quenching Means Lower Yields: Electrostatic Control of Cage Escape

**DOI:** 10.1021/acsphyschemau.5c00103

**Published:** 2025-12-29

**Authors:** Alberto Bianco, Mirco Natali, Giacomo Bergamini

**Affiliations:** † Department of Chemistry “Giacomo Ciamician”, 9296 University of Bologna, Via Piero Gobetti 85, 40129 Bologna, Italy; ‡ Department of Chemical, Pharmaceutical and Agricultural Sciences, University of Ferrara, Via Luigi Borsari 46, 44121 Ferrara, Italy

**Keywords:** Charge Recombination, Charge Transfer, Quenching, Photosensitizer, Quantum Yield

## Abstract

Photoredox catalysis often relies on excited-state quenching data to rationalize performance, yet such metrics can obscure the impact of solvent cage escape on overall efficiency. We report a systematic study of the effect of electrostatic interactions on the excited-state quenching, cage escape, and back-electron transfer processes in a benchmark system comprising methyl viologen (MV^2+^) and differently carboxylated ruthenium polypyridyl complexes with net charges from 2+ to 4–. Increasing electrostatic attraction between photosensitizer and MV^2+^ enhances the quenching rate constant (*k*_*q*_) up to the diffusion limit but simultaneously suppresses cage escape quantum yields, resulting in an inverse correlation between *k*_*q*_ and photochemical MV^•+^ production. Transient absorption spectroscopy confirms that cage escape, rather than quenching or back-electron transfer, governs the quantum yield of product formation. Protonation of carboxylate groups to yield uniformly 2+ complexes equalizes quenching rates and substantially increases cage escape efficiency for the originally anionic species. These results establish electrostatic control of charge separation as a decisive factor in photoredox catalysis and challenge the practice of predicting yields solely from quenching experiments. Consideration of both the initial and post-electron-transfer charges of the photocatalyst/quencher pair emerges as a general design principle for maximizing cage escape and, consequently, photoredox reaction efficiency.

## Graphical abstract

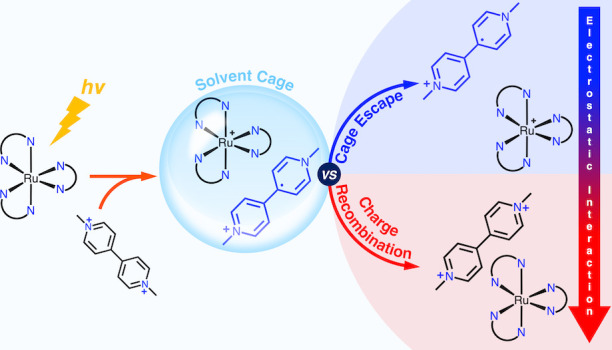

## Introduction

Photoredox catalysis harnesses light energy to drive chemical transformations by converting photons into redox equivalents via photosensitizer-initiated electron transfer.^1–3^ This approach enables reactions under significantly milder conditions than traditional thermal pathways, establishing photocatalysis as a powerful methodology in synthetic chemistry,^4,5^ as proved by the number of publications in this field, as depicted in Figure 1.

**Figure 1:**
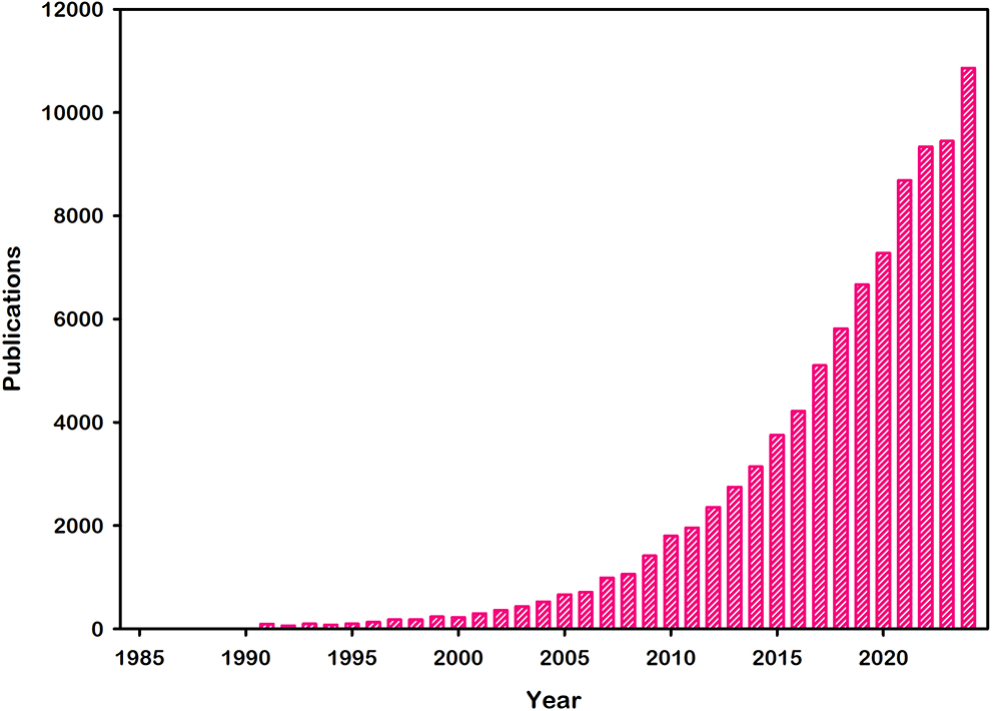
Annual number of publications containing the keyword *photocatalysis* from 1985 to 2024. Total number of publications 91013 (retrieved from *Web of Science Core Collection*).

However, a significant limitation in mechanistic studies is the reliance on luminescence quenching experiments to infer reaction routes.^6^ Frequently, the extent of photocatalyst quenching is directly correlated to product formation and reaction yield.^7–10^ While this correlation is frequently observed, it often oversimplifies the true mechanistic pathways. The common assumption that every quenched excited state productively generates a useful redox pair (an oxidized/reduced quencher and its corresponding photosensitizer) can lead to erroneous conclusions.^11–14^ Consequently, a more critical and thorough investigation is essential before making assertions in this area of study.^15–19^

As introduced in 1934 by Franck and Rabinowitsch, the primary products of photoinduced electron transfer are initially confined within a solvent cage,^20,21^ necessitating their escape for subsequent productive chemical reactions. Critically, charge recombination can spontaneously occur within this cage, preventing product accumulation despite luminescence quenching is observed.^22,23^ While photoredox catalysis has traditionally been dominated by synthetic development, often overlooking the photophysical and photochemical background and specifically the cage escape step, its importance for overall reaction outcomes is now gaining significant recognition.^14,15,24–33^

In 1972 Lorand coined the term “Cage Effect”,^34^ and since then numerous factors influencing cage escape have been extensively investigated, with particular attention paid to the effects of ionic strength and counterions,^35^ as highlighted by many works by Hoffman and co-workers.^23,36–42^

Furthermore, multiple other parameters, including heavy atom effect,^43–45^ spin and magnetic field effects,^46^ solvent polarity and viscosity,^47^ temperature,^36^ distance and noncovalent interactions among system components,^14,24,48,49^ can also dramatically affect the cage escape process.

Despite these well-known insights, the incomplete integration of these critical considerations throughout the evolution of photocatalysis has frequently resulted in flawed interpretations and data, making cage escape quantum yield an extremely difficult to predict value.^50^

Building on these concepts, Meyer, Troian-Gautier, and their colleagues extensively investigated the impact of cage escape on various photochemical reactions. Their work demonstrated the crucial role this step plays in determining reaction yields, as documented in numerous publications,^28,30,31,51–57^ demonstrating the direct correlation between cage escape and reaction quantum yields.^12^ Additionally, these findings were recently summarized in a comprehensive review, which not only presented the current state of knowledge but also offered valuable guidance for future research directions.^24^

In a recent paper, Wenger and co-workers highlighted this direct correlation in three benchmark photoredox reactions.^14^ These reactions were performed with various electron donors, using either [Ru­(bpz)_3_]^2+^ or [Cr­(dqp)_2_]^3+^ as the photosensitizer, observing higher reaction quantum yields using the ruthenium-based photosensitizer.

The study shows that for Ru­(II) the in-cage charge recombination is more exergonic than for Cr­(III), placing it deeper into the Marcus inverted region. This slows recombination, increases cage escape efficiency, and boosts reaction yield, highlighting how the choice of photocatalyst critically determines yields by governing in-cage recombination rates.

Among many investigated parameters, surprisingly, the electrostatic charges of the two pristine main components within a photoredox system have been poorly explored.^14,24,58^ This oversight means their influence on not only the cage escape efficiency, but also the other two steps involved in a photoinduced electron transfer system, specifically the excited-state quenching and the *back-electron transfer* (BeT) processes, remains underexamined, highlighting how systematic analysis of these aspects can rationalize some behaviors of these systems.

An intuitive initial assumption might suggest that electrostatic attraction between the system components would prompt a more efficient interaction between the excited-state photocatalyst and the quencher, thus improving photon-to-redox conversion. However, this simplification overlooks the crucial role of the cage escape step, which significantly influences the overall process efficiency.

In this context, the present study aims to elucidate the discrepancies among excited-state quenching efficiencies, the extent of cage escape, and the back-electron transfer pathway of escaped products.

Utilizing the most widely studied photoinduced electron transfer system,^23,24,37,39,42,59–62^ we conducted a systematic analysis of the dynamic quenching of a series of ruthenium polypyridyl complexes, with charges from 2+ to 4–, by the electron acceptor methyl viologen dication (MV^2+^). We subsequently determined the cage escape quantum yield for each photosensitizer–electron acceptor pair. Finally, we investigated the back-electron transfer process that follows cage escape, focusing on the diffusion-controlled reaction between the photogenerated methyl viologen radical cation (MV^•+^) and the oxidized ruthenium species. Scheme 1 provides a comprehensive overview of the photophysical and photochemical processes investigated in this study. It distinctly illustrates the key mechanistic steps, emphasizing the crucial difference between the in-cage charge recombination, henceforth referred to as ″charge recombination″ for clarity throughout the paper, and the back-electron transfer step. This distinction is critical because in-cage recombination occurs immediately following the photoinduced charge separation within the solvent shell, while back-electron transfer involves different molecular species after they have successfully escaped the solvent cage.

**Scheme 1:**
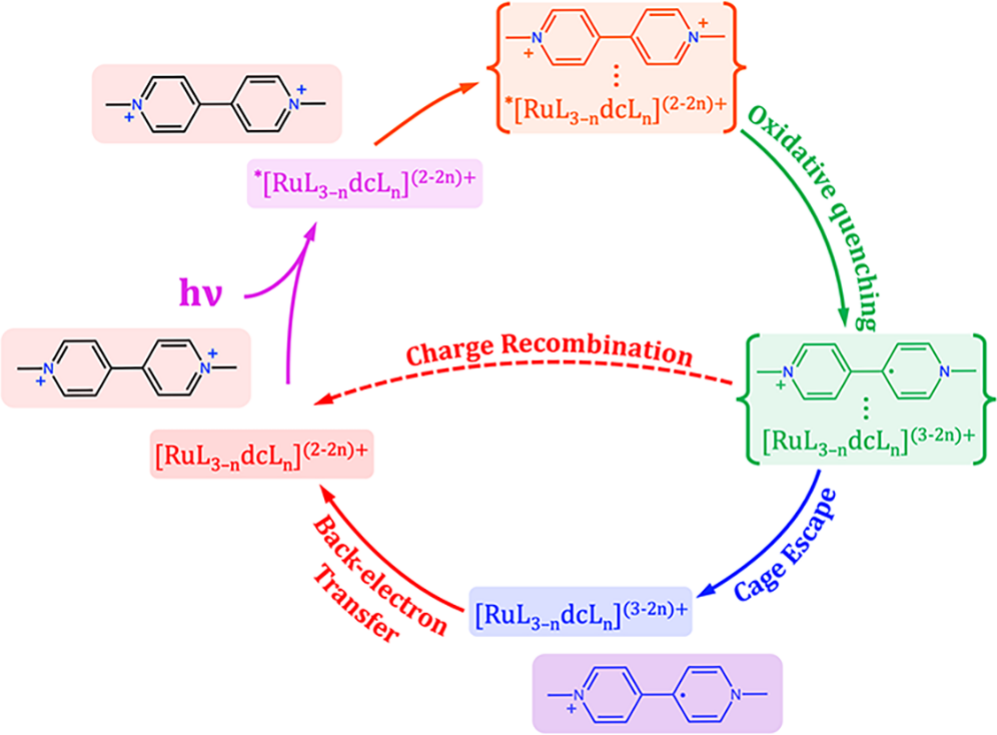
Representation of the Photoinduced Processes Investigated in the Present Study

## Methods

### Chemicals

High-purity triethanolamine (TEOA, ≥ 99.0%), fuming hydrochloric acid (37 wt %), and sodium hydroxide (≥99.9%) were purchased from Merck and used with no further purification.

Tris­(2,2′-bipyridyl)­ruthenium­(II) chloride hexahydrate ([Ru­(bpy)_3_]­Cl_2_·6H_2_O, 99.95%) was purchased from Merck and recrystallized tree times from methanol. Bis­(2,2′-bipyridyl)­(2,2′-bipyridyl-5,5′-dicarboxylic acid) ruthenium­(II) chloride ([Ru­(bpy)_2_(H_2_dcbpy)]­Cl_2_), (2,2′-bipyridyl) bis­(2,2′-bipyridyl-5,5′-dicarboxylic acid) ruthenium­(II) chloride ([Ru­(bpy)­(H_2_dcbpy)_2_]­Cl_2_), and Tris­(2,2′-bipyridyl-5,5′-dicarboxylic acid) ruthenium­(II) chloride ([Ru­(H_2_dcbpy)_3_]­Cl_2_) were synthesized and purified according to literature procedures.^63^

1,1′-Dimethyl-4,4′-bipyridinium dichloride (MVCl_2_, > 98%) was purchased from Merck and recrystallized tree times from ethanol. Argon used for purging (filtered on Drierite, 99.9995% purity) was supplied by SIAD. Type 1 ultrapure water was obtained with a Sartorius Arium Comfort II apparatus.

### Spectroscopic Methods

UV/Visible absorption spectra were recorded on a PerkinElmer λ45 or Agilent Cary 300 double beam spectrophotometers, using custom-made quartz gastight cuvette with 1.00 cm optical path length.

Emission spectra were recorded on an Edinburgh Instruments FS5 equipped with a Hamamatsu R13456 photomultiplier tube; using custom-made quartz gastight cuvette with 1.00 cm path length.

Emission lifetime decays were recorded on an Edinburgh Instruments FLS920 spectrofluorometer equipped with a TCC2 electronic module for time-correlated single photon counting data acquisition (305 fs resolution), a PicoQuant LDH–P-C-405 pulsed diode laser and a Hamamatsu H5773–04 metal package photomultiplier tube; using custom-made quartz gastight cuvette with 1.00 cm path length. All the decays were fitted with monoexponential functions.

Transient absorption decays were recorded on an Edinburgh Instruments LP980 ns transient absorption spectrometer (800 ps resolution) equipped with a Hamamatsu R928 photomultiplier and a Litron Nano Nd:YAG pulsed laser (5 Hz) with third harmonic generator (λ_exc_ = 355 nm).

### Irradiation Methods

All 2.0 mL samples, previously purged with argon, were irradiated for a total time of 10 min under vigorous magnetic stirring. The light source utilized was a high-power light-emitting diode (LED Engin LuxiGenTM LZ1–10B202–0000, 460 nm), operated at a constant current of 50 mA and positioned at a fixed distance of 10.0 cm from the sample within a custom-made quartz gastight cuvette (1.00 cm path length).

## Results

As previously described, photoexcitation in these systems initiates several key processes: excited-state quenching (specifically, by an electron acceptor), cage escape, and back-electron transfer between the reduced electron acceptor and the oxidized photocatalyst. By employing a combination of steady-state and time-resolved spectroscopic techniques, with appropriate experimental adjustments, each of these three processes can be investigated independently, and by combining all these techniques it is possible to understand the complete photochemical pathway. Recently, Connell and co-workers proposed that steady-state in operando measurements can yield information comparable to time-resolved methods, offering a simpler alternative for probing photochemical systems.^26^

For clarity, the subsequent results presentation and discussion is organized into distinct sections, each dedicated to each of the photoinduced processes described above.

### Excited-State Quenching

The impact of electrostatic charge of the photosensitizer on the excited-state quenching mechanism was explored by employing a series of analogous ruthenium­(II) polypyridyl complexes. For a systematic investigation of complex net charges, we chose three complexes with varying numbers of carboxylate groups, alongside the well-known [Ru­(bpy)_3_]^2+^, as detailed in Scheme 2.

**Scheme 2:**
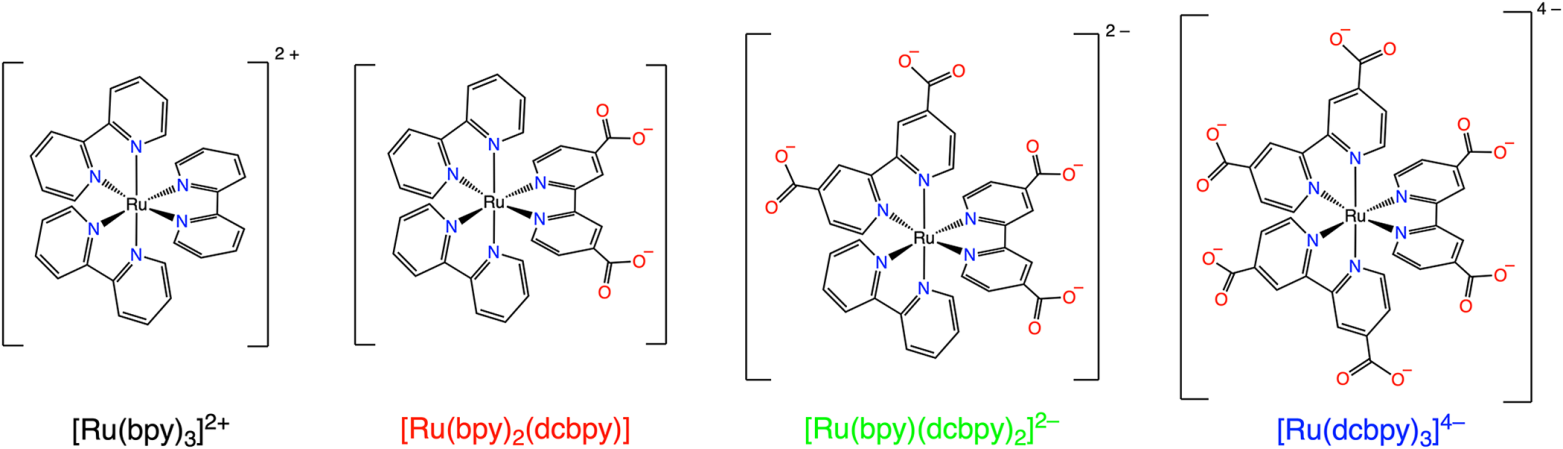
Chemical Structure of the Four Ruthenium Complexes Employed in the Present Study

Our study specifically investigates how the total charge of the photosensitizer influences photoinduced bimolecular processes involving a dicationic electron acceptor. Therefore, to guarantee complete deprotonation of the carboxylate groups and ensure defined charge states for the complexes, all subsequent experiments were performed in alkaline media. A freshly prepared 0.1 mM NaOH aqueous solution was utilized for this purpose, checking the pH prior and after all the measurements on the sample (pH = 10.00 ± 0.05).

As can be seen in Figure 2, these complexes exhibit similar absorption and emission spectra, together with analogous photophysical and electrochemical properties (detailed in Table S1).^63^ For this reason, the four complexes employed allow for a comparative analysis where contributions from factors other than electrostatic charge, specifically reaction ΔG°, steric effects, and diffusion rate, can be considered minor factors in their bimolecular photoinduced electron transfer processes.

**Figure 2:**
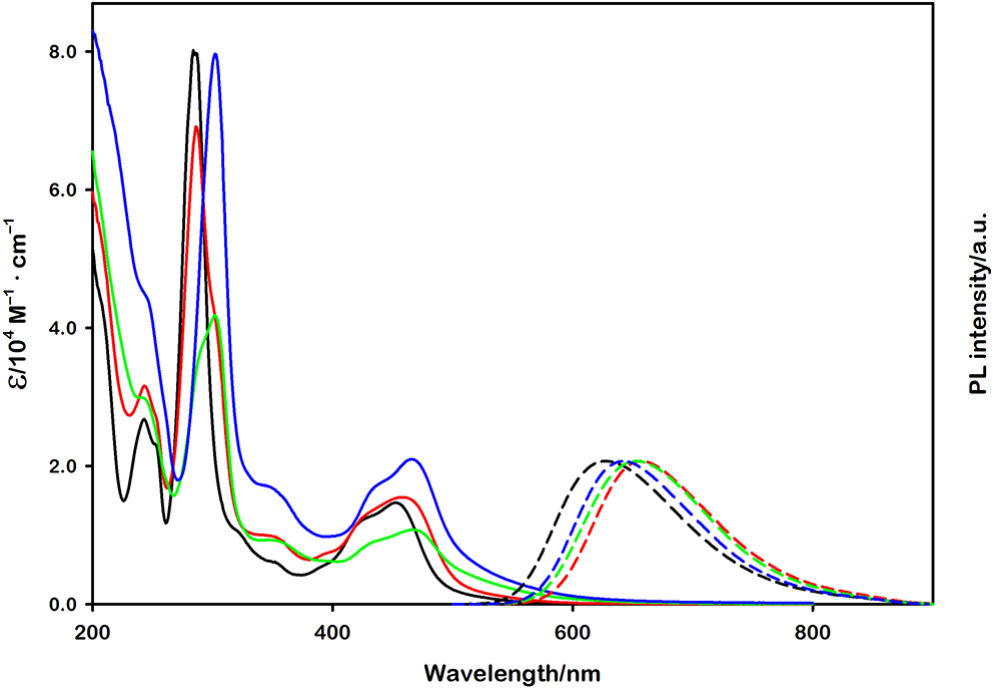
Absorption (solid lines) and emission (dashed lines) spectra of [Ru­(bpy)_3_]^2+^ (black), [Ru­(bpy)_2_(dcbpy)] (red), [Ru­(bpy)­(dcbpy)_2_]^2–^ (green), and [Ru­(dcbpy)_3_]^4–^ (blue). All the spectra were recorded in 0.1 mM NaOH aqueous solution (pH = 10.00).

To estimate the interaction between the complexes excited state and the electron acceptor, we subsequently conducted Stern–Volmer analysis for each complex, employing methyl viologen (MV^2+^) as the excited-state quencher. The choice of methyl viologen was primarily dictated by its common application in quenching studies, particularly involving ruthenium complexes.^42^ In addition to this, methyl viologen is initial a dicationic species, which results in a positively charged radical cation (MV^•+^) upon reduction. This characteristic is useful for our purposes, as we can exploit electrostatic interactions also in the products of the photoinduced electron transfer, as detailed in Table 1.

**Table 1:** Electrostatic Charge (Q) of the Electron Donor (Ru Complex) and Electron Acceptor (MV^2+^) Pairs before and after the Photoinduced Electron Transfer

Photosensitizer	Quencher	Q before eT	Q after eT
[Ru(bpy)_3_]^2+^	MV^2+^	2+/2+	3+/+
[Ru(bpy)_2_(dcbpy)]	MV^2+^	0/2+	+/+
[Ru(bpy)(dcbpy)_2_]^2–^	MV^2+^	2–/2+	–/+
[Ru(dcbpy)_3_]^4–^	MV^2+^	4–/2+	3–/+

Given the theoretical framework, we anticipated a direct correlation between the strength of the electrostatic interaction within the systems and their corresponding quenching efficiencies. To test this hypothesis, we performed a series of measurements which show a clear enhancement in quenching efficiency for systems with stronger electrostatic interactions, as detailed in Figure 3.

**Figure 3:**
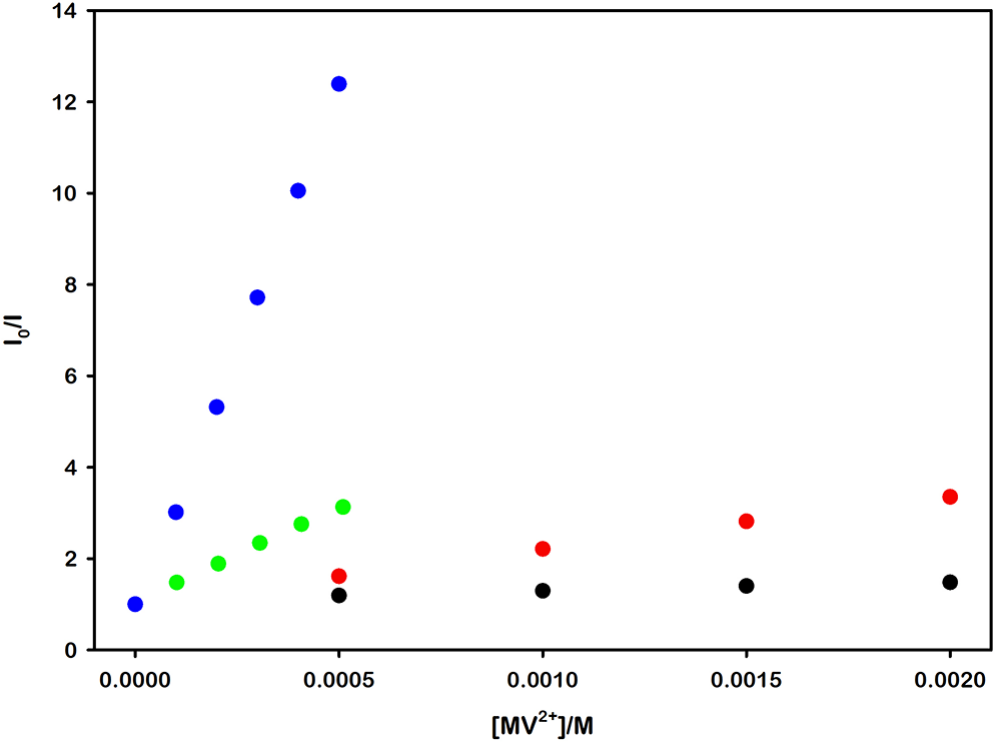
Steady-state emission Stern–Volmer plots illustrating the quenching by MV^2+^ of *­[Ru­(bpy)_3_]^2+^ (black dots), *­[Ru­(bpy)_2_(dcbpy)] (red dots), *­[Ru­(bpy)­(dcbpy)_2_]^2–^ (green dots), and *­[Ru­(dcbpy)_3_]^4–^ (blue dots) in air-equilibrated 0.1 mM NaOH aqueous solution (pH = 10.00).

Specifically, while the quenching of the complexes excited-state by methyl viologen remain a dynamic process (see Figure S6 and Table S3), the electrostatic attraction between these two components significantly enhances the quenching efficiency, as evidenced by the steeper slopes observed in the Stern–Volmer plots, and the consequent increase in the Stern–Volmer constant *K*_*SV*_ and the bimolecular quenching rate constant *k*_*q*_, listed in Table 2.[Fn fn1] ^†^

**Table 2:** Ru Complex Lifetimes, and Stern–Volmer and Quenching Constants for the Ru–MV^2+^ Pairs Investigated in Air-Equilibrated 0.1 mM NaOH Aqueous Solution (pH = 10.00) with Steady-State Emission Techniques

Photosensitizer	τ_0_/ns (AE)	*K*_*SV*_/M^–1^	*k*_q_/M^–1^ · s^–1^
[Ru(bpy)_3_]^2+^	372	111	3.0 · 10^8^
[Ru(bpy)_2_(dcbpy)]	380	916	2.4 · 10^9^
[Ru(bpy)(dcbpy)_2_]^2–^	345	4 183	1.0 · 10^10^
[Ru(dcbpy)_3_]^4–^	439	15 620	3.6 · 10^10^

Although a higher quenching rate suggests that the hexacarboxylated ruthenium complex, [Ru­(dcbpy)_3_]^4–^, should be a more efficient photocatalyst for the photoreduction of methyl viologen, this conclusion is based solely on a single kinetic parameter. To confirm this assumption, a comprehensive evaluation of the photocatalytic performance would require further investigation, specifically investigating the photochemical reaction kinetic.

### Photochemical MV^•+^ Production

Following the standard approach for photocatalysis studies, we evaluated the activity of these photocatalysts by directly performing the photochemical reaction of interest. Specifically, we monitored the formation of reduced methyl viologen (MV^•+^) formation under irradiation. The reaction solutions contained one of the ruthenium complexes, MV^2+^ as an electron acceptor, and *triethanolamine* (TEOA) as an electron source, closing the photochemical cycle and allowing the irreversible accumulation of MV^•+^ (Scheme 3).

**Scheme 3:**
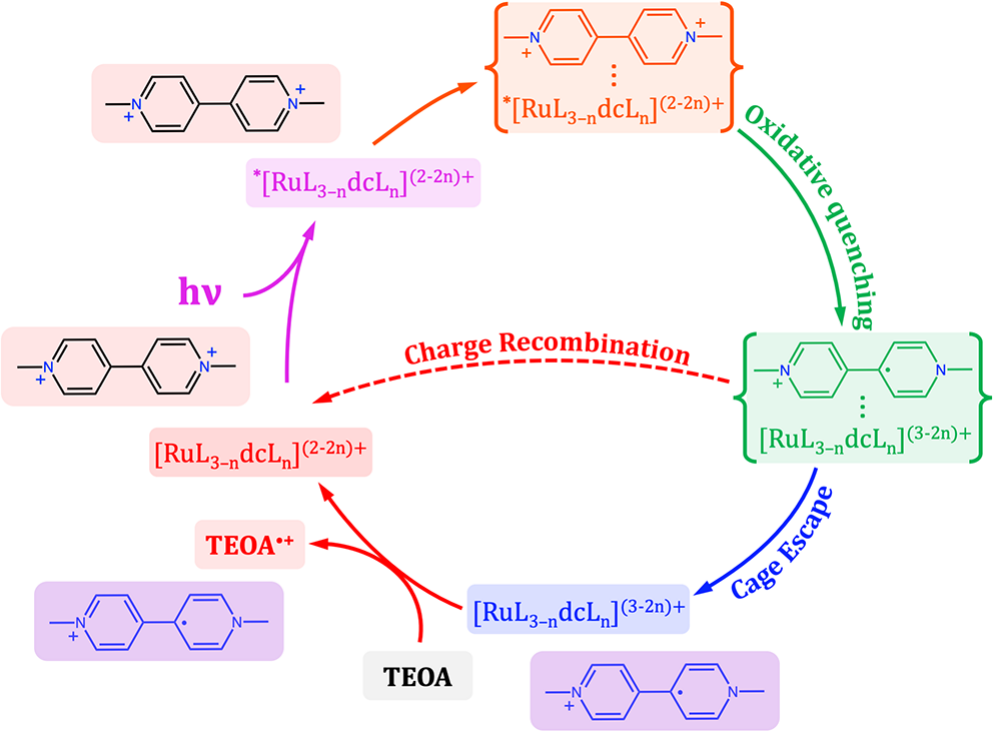
Representation of the Photochemical Cycle Used to Reduce and Accumulate Methyl Viologen

A rigorous comparative analysis of the four photocatalytic systems necessitated specific experimental adjustments. Then, all the anaerobic solutions of the complexes were irradiated at λ_irr_ = 460 nm, with Ru concentrations adjusted to ensure equal absorbance at this wavelength. The methyl viologen concentration added to each solution was in the right amount to quench 50% of the excited states. By standardizing the conditions across all four systems, we were able to directly measure and compare their relative reaction quantum yields, thereby ensuring that all subsequent measurements were relative to one another. The MV^•+^ amount produced during irradiation was determined by Beer–Lambert-Bouguer law, measuring the variation of the absorption at 605 nm (MV^•+^ ε_605 nm_ = 13 700 M^–1^ cm^–1^),^64^ obtaining the results depicted in Figure 4 (see SI for comprehensive data).

**Figure 4:**
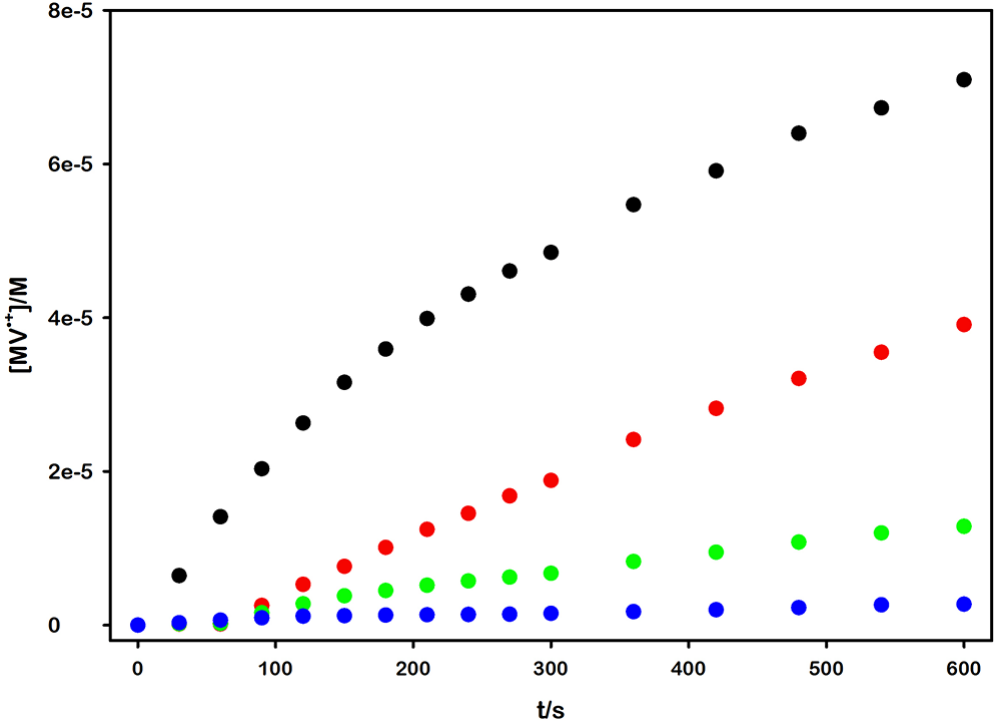
Methyl viologen radical cation MV^•+^ concentration obtained during 460 nm irradiation of the reaction mixtures containing TEOA 0.1 M (pH = 10.50), [Ru­(bpy)_3_]^2+^ and 5.6 mM MV^2+^ (black dots), [Ru­(bpy)_2_(dcbpy)] and 0.8 mM MV^2+^ (red dots), [Ru­(bpy)­(dcbpy)_2_]^2–^ and 172.4 μM MV^2+^ (green dots), [Ru­(dcbpy)_3_]^4–^ and 40.7 μM MV^2+^ (blue dots).

A huge discrepancy was observed between the excited-state quenching and the ultimate product yield. Specifically, the extent of viologen reduction upon irradiation exhibited an inverse correlation with the observed quenching rate constant *k*_*q*_. Notably, this counterintuitive behavior was more evident with the hexacarboxylated complex (with a total charge of 4−), which, despite demonstrating the highest *k*_*q*_ among all complexes tested, yielded a negligible amount of photogenerated viologen radical cation. This finding is a critical consideration for photocatalytic system design, as it highlights that a high quenching constant, while indicative of efficient electron-transfer, does not inherently guarantee high product yields.

### Cage Escape Quantum Yields

The observed discrepancies provide compelling evidence that quenching efficiency alone may not be a reliable metric for understanding complex photocatalytic mechanisms and can lead to oversimplified or erroneous conclusions. As previously stated, a fundamental requirement for productive photocatalysis is the efficient separation of photogenerated electron-transfer products, which must successfully escape the solvent cage to prevent rapid geminate charge recombination. In the absence of a completely closed photochemical cycle, these products exist as transient species within the reaction medium. Their limited lifetime, governed by back-electron transfer or other deactivation pathways, ultimately hinders the accumulation of the desired product and compromises overall catalytic efficiency, rather than participating in the intended cascade of reactions.

To pinpoint the source of this behavior, we employed flash photolysis to directly monitor and quantify the transient formation of the methyl viologen radical cation MV^•+^ upon photoexcitation.

The quantum yield of MV^•+^ production *Φ*_*product*_ was then determined by comparing the measured MV^•+^ concentration with the number of absorbed photons (determined via transient actinometry, see SI for details). The calculation utilized the following equation, which is valid under the iso-absorbing conditions between the sample and the reference solutions.^54^

Φproduct=ΔAproductΔεproductΔAreferenceΔεreference·Φreference

The outcomes of the experiments for each sample are presented in Figure 5, which provides a visual summary of the four different systems investigated in this study.

**Figure 5:**
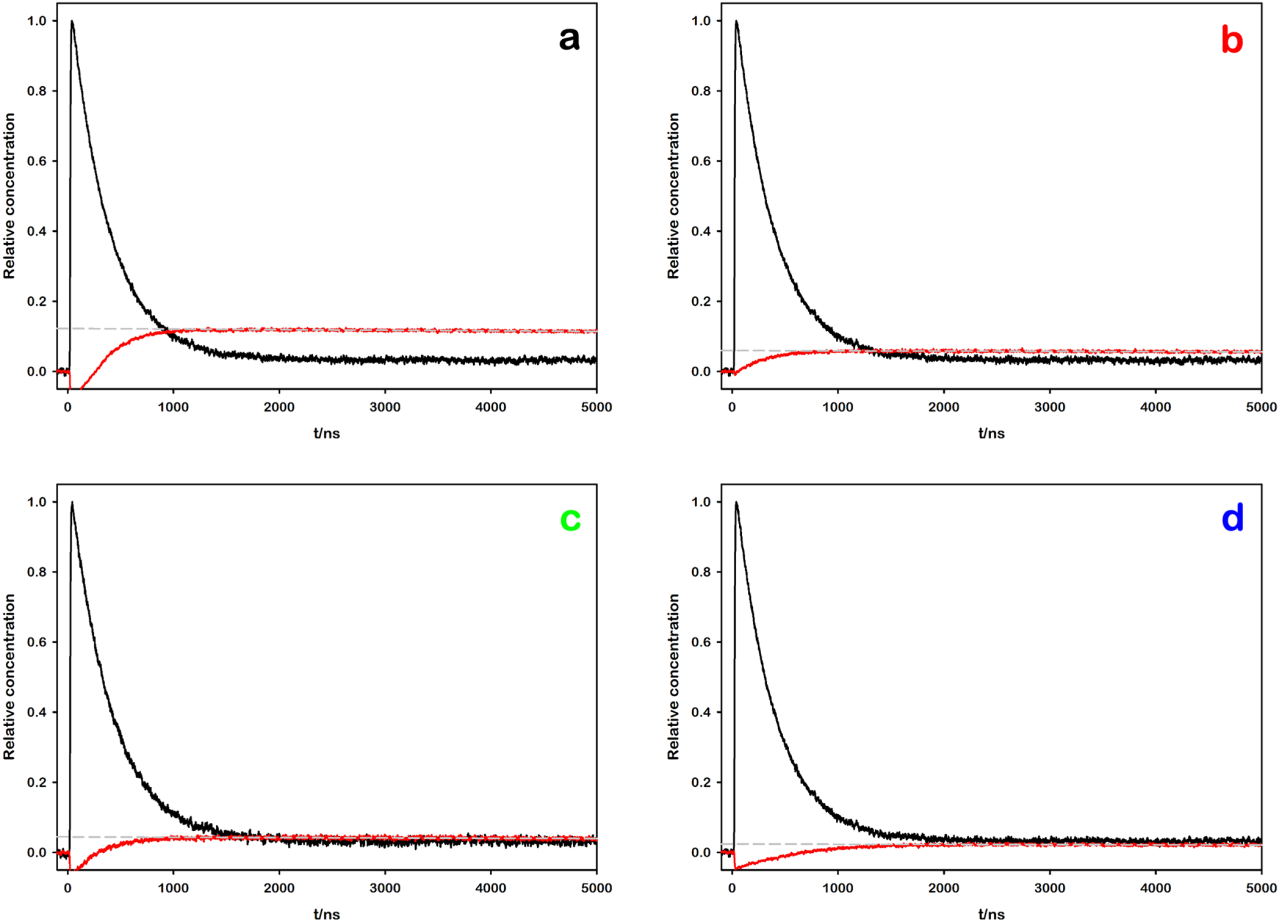
Transient absorption kinetic traces of the photoexcited samples (Ru–MV^2+^ in 0.1 mM NaOH, pH = 10.00) after laser flash photolysis (λ_exc_ = 355 nm). The black lines show the decay of the *­[Ru­(bpy)_3_]^2+^ at 450 nm, used as transient actinometer. The red lines display the growth of MV^•+^ for (a) [Ru­(bpy)_3_]^2+^, (b) [Ru­(bpy)_2_(dcbpy)], (c) [Ru­(bpy)­(dcbpy)_2_]^2–^, and (d) [Ru­(dcbpy)_3_]^4–^ as the photosensitizer. The gray dashed lines represent the linear fittings of the 605 nm kinetic traces, used to extrapolate relative MV^•+^ concentration at *t* = 0.

Since methyl viologen radical cation is the product of a dynamic bimolecular process, *Φ*_*product*_ depends on the fraction of quenched excited state. By adjusting *Φ*_*product*_ based on the number of quenched excited state, the Cage Escape Quantum Yield *Φ*_*CE*_ was determined according to the following equation:^24,54,65^

ΦCE=ΦproductQuenchedESfraction=Φproduct(1−ττ0)

A comprehensive summary of the experimental results is provided in Table 3 below.

**Table 3:** MV^•+^ Production and Cage Escape Quantum Yields for the Ru–MV^2+^ Investigated Pairs

Photosensitizer	*Φ*_*Prod*_	1 – τ/τ_0_	*Φ*_*CE*_
[Ru(bpy)_3_]^2+^	0.123	0.580	0.212
[Ru(bpy)_2_(dcbpy)]	0.060	0.485	0.124
[Ru(bpy)(dcbpy)_2_]^2–^	0.044	0.517	0.085
[Ru(dcbpy)_3_]^4–^	0.024	0.351	0.068

As detailed in Table 3, the cage escape quantum yield for the benchmark [Ru­(bpy)_3_]^2+^–MV^2+^ system is consistent with previously published data.^24^ In contrast, the other systems under investigation exhibited a notably lower *Φ*_*CE*_. This reduced cage escape efficiency provides a crucial explanation for the observed behavior in the MV^•+^ photoaccumulation experiments.

### Back-Electron Transfer Process

Upon photoexcitation and subsequent oxidative quenching of the excited state, the initial photochemical event yields a geminate pair consisting of the oxidized ruthenium complex and the reduced methyl viologen radical cation. Following successful cage escape, these two photogenerated species are released from the solvent cage and diffuse independently into the bulk solution.

In the absence of an electron donor, back-electron transfer will inevitably occur to restore the pristine ruthenium complex. This process, which involves the reduced viologen donating an electron back to the oxidized ruthenium, effectively regenerates the initial system. The disappearance of the transient species after the laser pulse can be monitored on a time scale of hundreds of microseconds. Table 4 reports the bimolecular rate constant that can be estimated by plotting the reciprocal of the recorded kinetic traces and applying a second-order kinetic treatment (see Figure S18).^66,67^

**Table 4:** Bimolecular Rate Constant of the Back-Electron Transfer Process between the Oxidized Ruthenium Complexes and the Reduced Methyl Viologen MV^•+^ Investigated Pairs

Oxidized complex	Reduced viologen	*k*_*BeT*_/M^–1^·s^–1^
[Ru(bpy)_3_]^3+^	MV^•+^	7.8 · 10^9^
[Ru(bpy)_2_(dcbpy)]^+^	MV^•+^	1.8 · 10^10^
[Ru(bpy)(dcbpy)_2_]^−^	MV^•+^	2.9 · 10^10^
[Ru(dcbpy)_3_]^3–^	MV^•+^	3.4 · 10^10^

The influence of electrostatic attraction on the back-electron transfer rate was observed to be analogous to its effect on excited-state quenching, with increased attraction leading to a faster rate. However, the magnitude of this effect on back-electron transfer is substantially less significant than its impact on quenching. This suggests that while it plays a role, its contribution to the final MV^•+^ production yield is limited. The cage escape of the charge-separated species, therefore, remains the dominant and rate-determining step in the overall process. This recombination pathway is typically considered negligible in photocatalytic mechanisms, as the presence of a high concentration of sacrificial electron or hole donor continuously regenerates the photocatalyst, thereby suppressing this particular deactivation process.^68^

### Effect of Protonation

As previously mentioned, the complexes investigated in this study exhibit pH-dependent charge states as a result of their intrinsic acid–base equilibria. By preparing all these complexes in a strongly acidic medium (10 mM HCl), the carboxylic acid groups appended to the bipyridyl ligands were fully protonated, which ensured that all four complexes adopted a uniform and well-defined 2+ electrostatic charge, as depicted in Scheme 4.

**Scheme 4:**
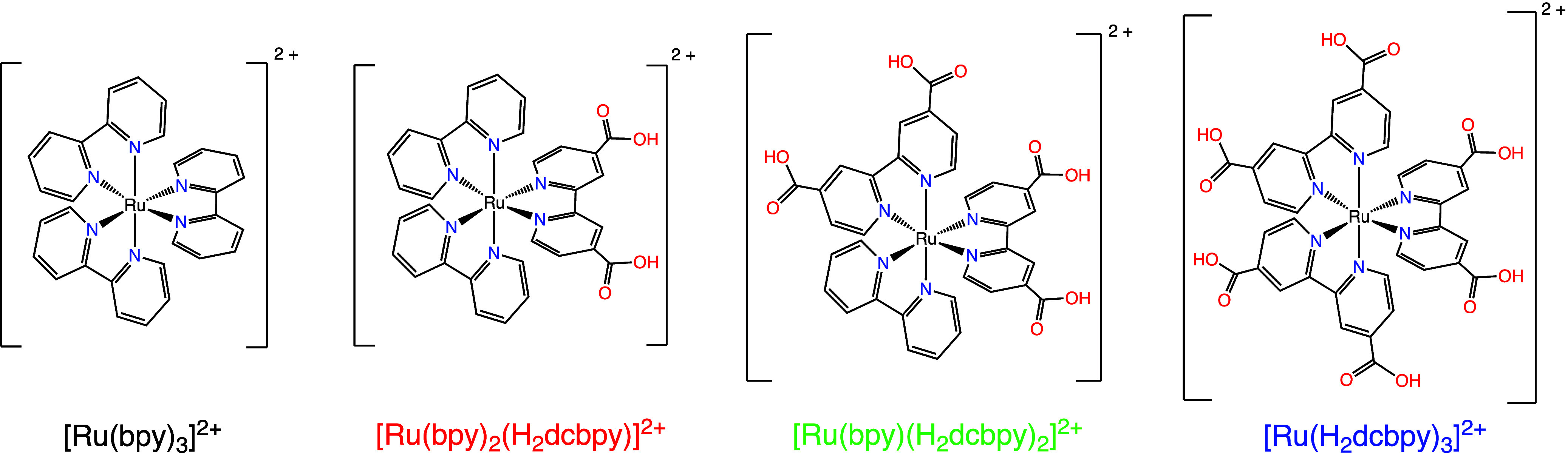
Chemical Structure of the Four Protonated Ruthenium Complexes

Based on the observed photochemical properties, we hypothesized that analogous complexes possessing an equivalent charge would exhibit similar behavior in the bimolecular processes described previously. To test this hypothesis, we systematically performed a series of Stern–Volmer quenching experiments on each of the protonated complexes, reported in Figure 6, allowing us to quantitatively assess and compare their excited-state deactivation pathways with the deprotonated counterparts.

**Figure 6:**
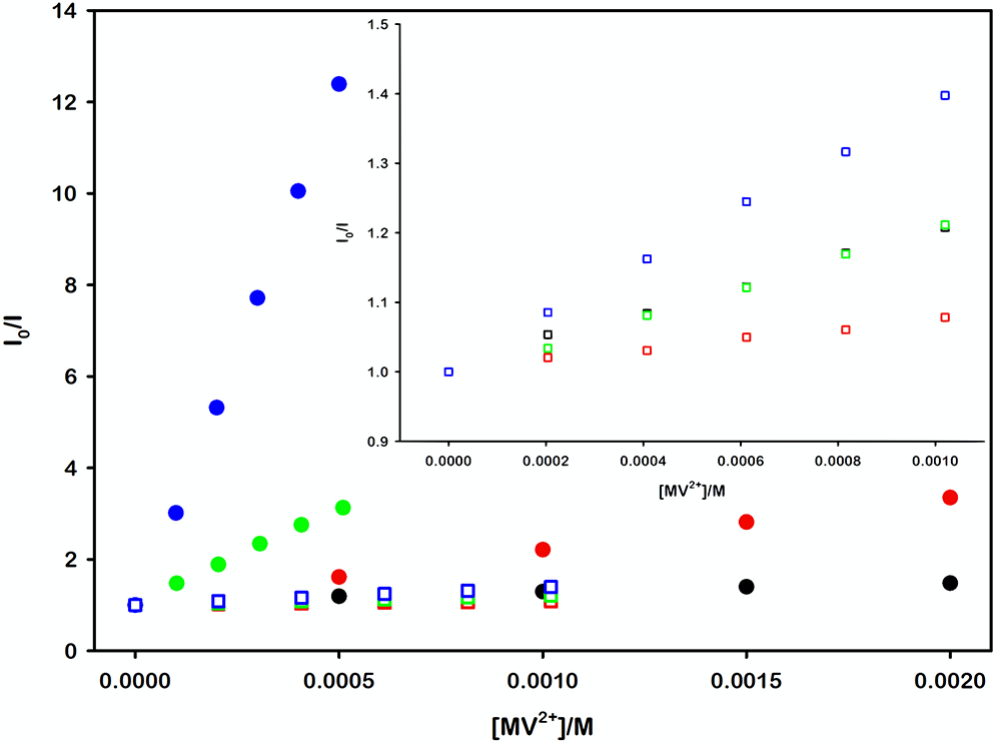
Comparison of the Stern–Volmer plots illustrating the quenching by MV^2+^ of *­[Ru­(bpy)_3_]^2+^ (black), *­[Ru­(bpy)_2_(dcbpy)] (red), *­[Ru­(bpy)­(dcbpy)_2_]^2–^ (green), and *­[Ru­(dcbpy)_3_]^4–^ (blue) in 0.1 mM NaOH aqueous solution (pH = 10.00, solid dots); and of *­[Ru­(bpy)_3_]^2+^ (black), *­[Ru­(bpy)_2_(H_2_dcbpy)]^2+^ (red), *­[Ru­(bpy)­(H_2_dcbpy)_2_]^2+^ (green), and *­[Ru­(H_2_dcbpy)_3_]^2+^ (blue) in 10 mM HCl aqueous solution (pH = 2.00, hollow squares). The upper inset shows the Stern–Volmer plots of the protonated complexes only.

The comparative analysis of the experimental data reveals that the protonation of the complexes yields remarkably similar quenching constants across all investigated samples, as Table 5 shows.

**Table 5:** Stern–Volmer and Quenching Constants for the Ru–MV^2+^ Pairs Investigated in 10 mM HCl Aqueous Solution (pH = 2.00)

Photosensitizer	*K*_*SV*_/M^–1^	*k*_*q*_/M^–1^ · s^–1^
[Ru(bpy)_3_]^2+^	200	5.4 · 10^8^
[Ru(bpy)_2_(H_2_dcbpy)]^2+^	80	3.4 · 10^8^
[Ru(bpy)(H_2_dcbpy)_2_]^2+^	210	6.1 · 10^8^
[Ru(H_2_dcbpy)_3_]^2+^	390	1.0 · 10^9^

This observation suggests a common quenching mechanism or a similar degree of interaction between the protonated complexes and the quencher. Furthermore, it is particularly noteworthy that the quenching efficiency of the [Ru­(bpy)_3_]^2+^–MV^2+^ system is almost unaffected by pH variations; the slight increase in K_SV_ arises from the higher ionic strength of the medium and is fully consistent with literature reports.^42^

We hypothesized that tuning the charge of the complex via the protonation of its carboxylic acid moieties could also significantly influence the subsequent steps of the photoinduced bimolecular process. To test this, we performed flash photolysis experiments on one of the protonated complexes, [Ru­(bpy)­(H_2_dcbpy)_2_]^2+^, depicted in Figure 7. The same suite of photophysical and photochemical parameters, which were previously determined for the deprotonated counterpart, were systematically measured and compared to isolate the specific effects of the charge modulation.

**Figure 7:**
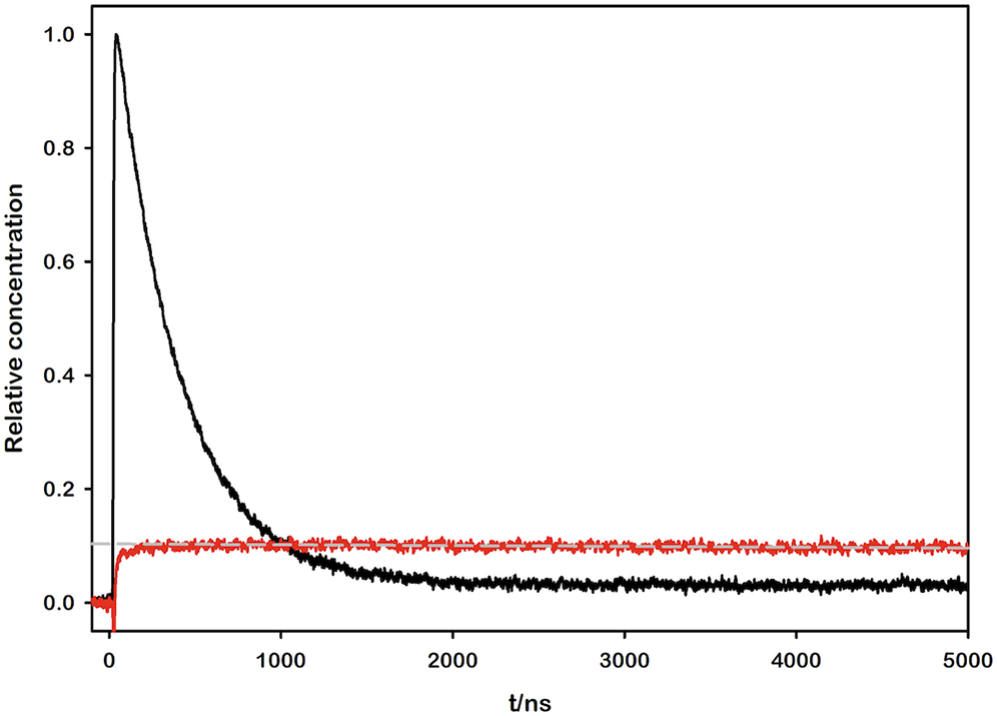
Transient absorption kinetic traces of the photoexcited sample (Ru–MV^2+^ in 10 mM HCl, pH = 2.00) after laser flash photolysis (λ_exc_ = 355 nm). The black line shows the decay of the *­[Ru­(bpy)_3_]^2+^ at 450 nm, used as transient actinometer. The red line displays the growth of MV^•+^ for [Ru­(bpy)­(H_2_dcbpy)_2_]^2+^ as the photosensitizer. The gray dashed line represents the linear fitting of the 605 nm kinetic trace, used to extrapolate relative MV^•+^ concentration at *t* = 0.

The results obtained from our investigation of this system are presented in Table 6. To highlight the impact of protonation, the table also includes a direct comparison with the data collected from the deprotonated system, thereby revealing significant differences in their photophysical and catalytic behavior.

**Table 6:** Excited-State Quenching, Cage Escape, and Back-Electron Transfer Parameters for the Methyl Viologen and Tetracarboxylated Ruthenium Polypyridyl Complex Pair Studied in 0.1 mM NaOH Aqueous Solution ([Ru­(bpy)­(dcbpy)_2_]^2–^, pH = 10.00) or in 10 mM HCl Aqueous Solution ([Ru­(bpy)­(H_2_dcbpy)_2_]^2+^, pH = 2.00)

Photosensitizer	Quencher	*K*_*SV*_/M^–1^	*k*_*q*_/M^–1^ · s^–1^	*Φ*_*CE*_	*k*_*BeT*_/M^–1^·s^–1^
[Ru(bpy)(dcbpy)_2_]^2–^	MV^2+^	4 183	1.0 · 10^10^	0.085	2.9 · 10^10^
[Ru(bpy)(H_2_dcbpy)_2_]^2+^	MV^2+^	210	6.1 · 10^8^	0.188	3.3 · 10^10^

It is noteworthy that the determined cage escape value for the protonated complex exhibits a strong similarity to the value reported for [Ru­(bpy)_3_]^2+^ (Table 3). This close correspondence provides compelling evidence that charge is the primary factor governing the efficiency of this cage escape process, suggesting that the electrostatic interactions between the charged species play a dominant role in preventing geminate recombination and promoting the separation of photogenerated radical pairs.

The minor variation in the back-electron transfer rate constants presented in Table 6 can be attributed to the interplay of two factors: ionic strength, which is known to accelerate the rate of dynamic processes,^42^ and the relative acidity of the oxidized complex, which can significantly modulate the acid–base equilibrium immediately following the photoinduced electron transfer event.

## Discussion

The role of electrostatic charges on a prototype photoinduced electron transfer process using a combination of ruthenium polypyridyl complexes with variable charges spanning from 2+ to 4– and MV^2+^ has been examined in detail through a combination of photochemical studies. Our data reveal that the charge of the ruthenium complex exerts a pronounced influence on both excited-state quenching dynamics and cage escape efficiency, with only a minor effect on the rate of back-electron transfer. This mechanistic picture implies that weaker electrostatic interactions between the excited-state and the quencher may reduce the quenching probability but simultaneously favor the dissociation of charge-separated pairs, thereby enhancing the probability of obtaining productive long-lived charge separation.

The results here presented highlight the limitations of using luminescence quenching data as a sole predictive measure of photocatalytic efficiency. In particular, the observed lack of correlation between the quenching constant and the overall quantum yield demonstrates that efficient dynamic quenching of the photoexcited ruthenium complex does not necessarily translate into productive charge separation. Instead, the inefficiency of charge-separated species to escape the solvent cage ultimately constrains the extent of viologen reduction.

Taken together, these findings underscore the pivotal role of electrostatics in governing the efficiency of photoinduced charge separation. By extracting the contributions of excited-state quenching, cage escape, and back-electron transfer, we demonstrate that electrostatic interactions, both in the initial state and after photoinduced electron transfer, serve as a key design parameter for optimizing photocatalyst–acceptor pairs in productive photoredox transformations. This would indeed help to maximize cage escape quantum yields, which in turn leads to enhanced photoredox reaction rates and superior overall quantum yields.

## Conclusions

Through the merging of luminescence and transient absorption spectroscopy experiments on a series of ruthenium polypyridyl complexes, we achieved a comprehensive understanding of the role of electrostatic interactions in photocatalytic systems. A central finding of this work is indeed the identification of the cage escape process as the primary determinant of both photoredox reaction rates and overall photoreaction yields. This emphasizes that rapid and efficient quenching is insufficient; instead, the ability of the charge-separated species to escape geminate recombination is paramount for productive catalysis. Our findings thus highlight a significant limitation in conventional photocatalytic studies: the common practice of relying solely on excited-state quenching efficiency can be misleading as this metric does not fully capture the complexity of the whole photoredox cycle. These are expected to provide a roadmap for developing more efficient photocatalytic systems by moving beyond simple quenching considerations to a more nuanced understanding of charge separation and stabilization.

## Supplementary Material



## Footnotes

a Since the processes studied in this work involve charged species, to obtain the diffusion limit can be determined using the Debye–Smoluchowski equation.^69^ Consequently, the calculated diffusion rate constants for charged species in aqueous media can exceed 10^10^ M^–1^ cm^–1^, a finding well-established in the literature.^70^

## Notes

Additional experimental details, materials, and methods; spectroscopic and electrochemical properties of the ruthenium complexes, time-resolved Stern–Volmer analysis, MV^•+^ photoaccumulation experiments, and transient absorption traces for back-electron transfer rate constants determination (PDF)
