# Supplementary material for: When Better Quenching Means Lower Yields: Electrostatic Control of Cage Escape
Source: ACS Phys Chem Au. 2025 Dec 29;6(1):185–95. doi: 10.1021/acsphyschemau.5c00103 (PMC12856654; doi:10.1021/acsphyschemau.5c00103)
Supplement: Supplementary file 1 [file pg5c00103_si_001.pdf]

Supporting Information for

When Better Quenching Means Lower Yields: Electrostatic Control of Cage Escape

Alberto Bianco,[*1] Mirco Natali,[2] and Giacomo Bergamini*[*1]

[1]Department of Chemistry "Giacomo Ciamician", University of Bologna, Via Piero Gobetti 85, 40129 Bologna, Italy

[2]Department of Chemical, Pharmaceutical and Agricultural Sciences, University of Ferrara, Via Luigi Borsari 46, 44121 Ferrara, Italy

Corresponding authors e-mail addresses: alberto.bianco5@unibo.it; giacomo.bergamini@unibo.it

SPECTROSCOPIC AND ELECTROCHEMICAL PROPERTIES OF THE RU COMPLEXES

Table S1. Main spectroscopic and electrochemical properties of the ruthenium complexes employed in this study.

Photosensitizer	$\lambda_{max\ MLCT}$ / nm	$\lambda_{max\ EM}$ / nm	τ / ns (AE)	τ / ns (Ar)	*E° / V vs NHE[a]
$[Ru(bpy)_3]^{2+}$	452	615	372	600	–0.86
$[Ru(bpy)_2(dcbpy)]$	458	642	380	513	–0.85
$[Ru(bpy)(dcbpy)_2]^{2-}$	468	635	345	580	–0.75
$[Ru(dcbpy)_3]^{4-}$	466	629	439	682	–0.81

[a] Retrieved from Reference S1

STERN-VOLMER ANALYSIS

To confirm that the quenching mechanism between the excited states of the complexes and methyl viologen is dynamic, we performed a time-resolved Stern-Volmer analysis, as described in the main text.

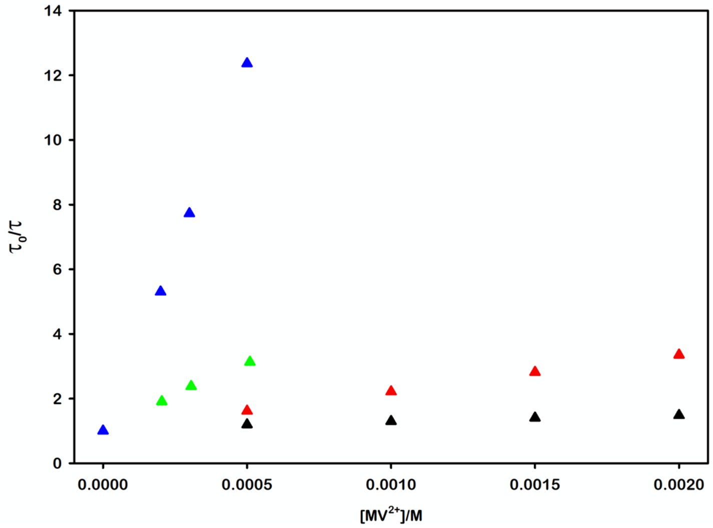

Figure S1. Time-resolved emission Stern-Volmer plots illustrating the quenching by MV^{2+} of $*[Ru(bpy)_3]^{2+}$ (black triangles), $*[Ru(bpy)_2(dcbpy)]$ (red triangles), $*[Ru(bpy)(dcbpy)_2]^{2-}$ (green triangles), and $*[Ru(dcbpy)_3]^{4-}$ (blue triangles) in air-equilibrated 0.1 mM NaOH aqueous solution (pH = 10.00).

A comparison of the Stern-Volmer and quenching rate constants obtained from both time-resolved and steady-state techniques revealed no significant differences (Table S2). This consistency confirms that the excited-state quenching process remains dynamic, even as the electrostatic interactions increase.

Table S2. Stern-Volmer and quenching constants for the Ru – MV^{2+} pairs investigated in 0.1 mM NaOH aqueous solution (pH = 10.00) with time-resolved emission techniques.

Photosensitizer	K_{SV} / M^{-1}	k_q / $M^{-1} \cdot s^{-1}$
$[Ru(bpy)_3]^{2+}$	110	$3.0 \cdot 10^8$
$[Ru(bpy)_2(dcbpy)]$	912	$2.4 \cdot 10^9$
$[Ru(bpy)(dcbpy)_2]^{2-}$	4 191	$1.0 \cdot 10^{10}$
$[Ru(dcbpy)_3]^{4-}$	15 600	$3.6 \cdot 10^{10}$

**$[Ru(bpy)_3]^{2+}$ – MV^{2+} Stern-Volmer Analysis*

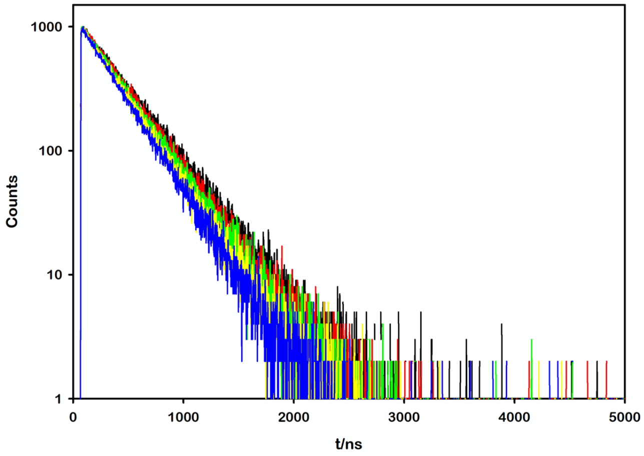

Figure S2. Luminescence decays of *$[Ru(bpy)_3]^{2+}$ with no MV^{2+} (black), with 0.5 mM (red), with 1.0 mM (green), with 1.5 mM (yellow), and with 2.0 mM (blue) MV^{2+} added in air-equilibrated 0.1 mM NaOH aqueous solution (pH = 10.00).

**[Ru(bpy)$_2$(dcbpy)] – MV^{2+} Stern-Volmer Analysis*

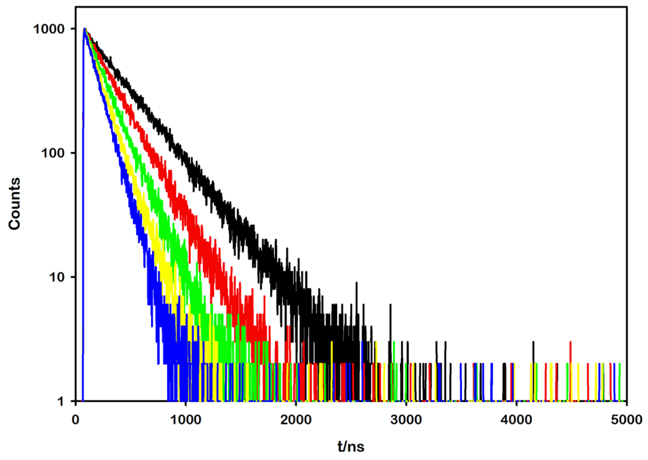

Figure S3. Luminescence decays of *[Ru(bpy)$_2$(dcbpy)] with no MV^{2+} (black), with 0.5 mM (red), with 1.0 mM (green), with 1.5 mM (yellow), and with 2.0 mM (blue) MV^{2+} added in air-equilibrated 0.1 mM NaOH aqueous solution (pH = 10.00).

**[Ru(bpy)(dcbpy)$_2$]$^{2-}$ – MV^{2+} Stern-Volmer Analysis*

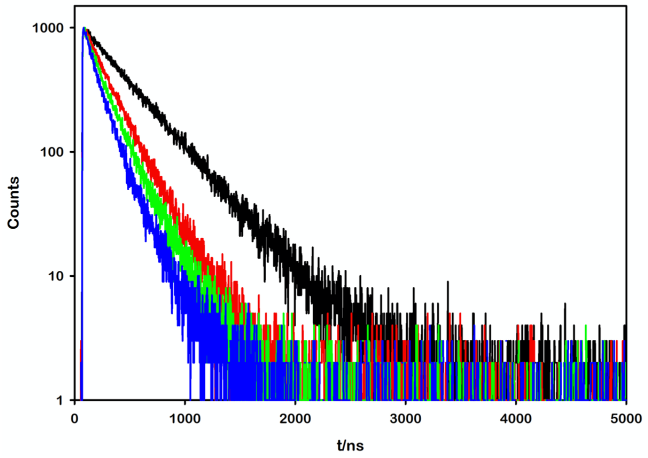

Figure S4. Luminescence decays of *[Ru(bpy)(dcbpy)$_2$]$^{2-}$ with no MV^{2+} (black), with 0.2 mM (red), with 0.3 mM (green), and with 0.5 mM (blue) MV^{2+} added in air-equilibrated 0.1 mM NaOH aqueous solution (pH = 10.00).

$*[Ru(dcbpy)_3]^{4-}$ – MV^{2+} Stern-Volmer Analysis

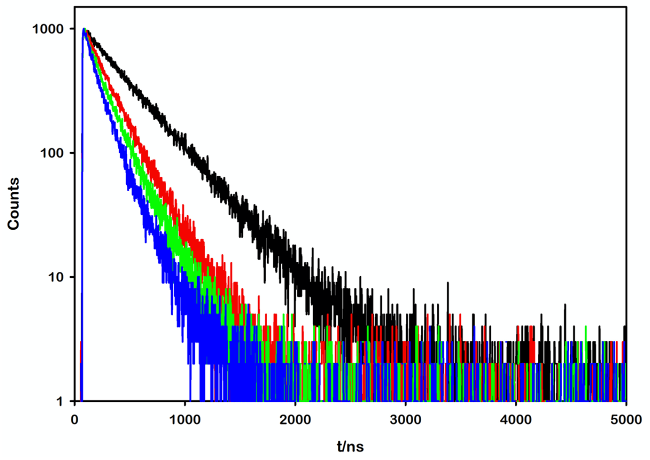

Figure S5. Luminescence decays of $*[Ru(dcbpy)_3]^{4-}$ with no MV^{2+} (black), with 0.2 mM (red), with 0.3 mM (green), and with 0.5 mM (blue) MV^{2+} added in air-equilibrated 0.1 mM NaOH aqueous solution (pH = 10.00).

Additionally, to ensure the observed quenching kinetics originated exclusively from dynamic mechanisms and not from static (ground-state) association between the anionic Ru species, as $[Ru(dcbpy)_3]^{4-}$, and the cationic quencher MV^{2+}, we performed a detailed Stern-Volmer investigation involving both steady-state and time-resolved emission techniques. Crucially, the protocol included varying the concentration of the Ru complex across three distinct samples, to which the same series of increasing MV^{2+} concentrations were subsequently added. The results as obtained are reported in Figure S6.

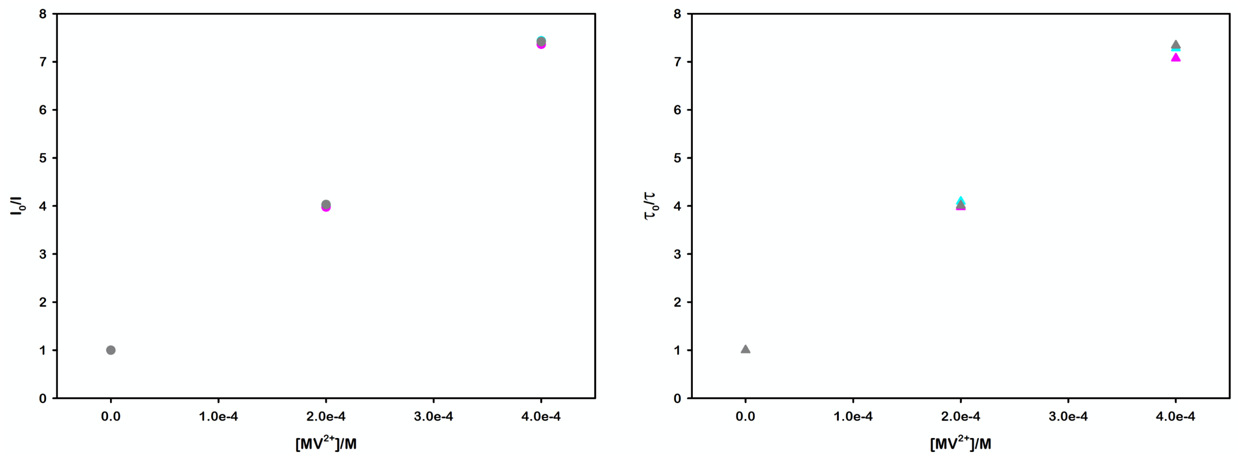

Figure S6. Steady-state (left side, circles) and time-resolved (right side, triangles) emission Stern-Volmer plots illustrating the quenching by MV^{2+} of different concentration $[Ru(dcbpy)_3]^{4-}$, specifically 36.2 μM (cyan), 10.7 μM (pink), and 1.1 μM (gray), in air-equilibrated 0.1 mM NaOH aqueous solution (pH = 10.00).

The quenching rate constants determined for the system under the above-described experimental conditions are comprehensively detailed in Table S3.

Table S3. Quenching constants for the $[Ru(dcbpy)_3]^{4-}$ – MV^{2+} pair investigated in air-equilibrated 0.1 mM NaOH aqueous solution (pH = 10.00) with steady-state and time-resolved emission techniques at different Ru concentration

$[[Ru(dcbpy)_3]^{4-}]$ / μM	$[MV^{2+}]_{final}$ / μM	k_q (SS) / $M^{-1} \cdot s^{-1}$	k_q (TR) / $M^{-1} \cdot s^{-1}$
36.2	400.0	$3.6 \cdot 10^{10}$	$3.5 \cdot 10^{10}$
10.7	400.0	$3.5 \cdot 10^{10}$	$3.4 \cdot 10^{10}$
1.1	400.0	$3.5 \cdot 10^{10}$	$3.5 \cdot 10^{10}$

Given that the ground-state preassociation mechanism is inherently and strongly dependent on the absolute concentrations of the photosensitizer and the quencher components, the striking consistency demonstrated by k_q across the examined $[Ru(dcbpy)_3]^{4-}$ concentration range serves as a crucial validation of the assumed dynamic mechanism, and provides compelling evidence for the effective absence of significant ground-state static interaction between the photosensitizer and the quencher.

Since the ground-state preassociation interaction is favored by strong electrostatic attraction, its absence in the highly charged 4–/2+ photosensitizer-quencher couple allows us to confidently and logically exclude the occurrence of such static complexation in the lower-charged 2–/2+ system. This collective evidence strongly suggests that the observed kinetic behavior in both systems is governed predominantly by dynamic, diffusion-controlled processes.

PHOTOCHEMICAL $MV^{\bullet+}$ PRODUCTION

For testing the photoreduction of the viologen for each ruthenium complex, we placed 2.0 mL of Ru complex in different concentrations to have the same 460 nm absorbance, MV^{2+} to quench 50% of the excited states and TEOA 0.1 M (pH = 10.50) in a custom-made gas-tight quartz cuvette (optical pathlength 1.00 cm). The samples were then deaerated vigorously bubbling argon through the solution for 15 minutes.

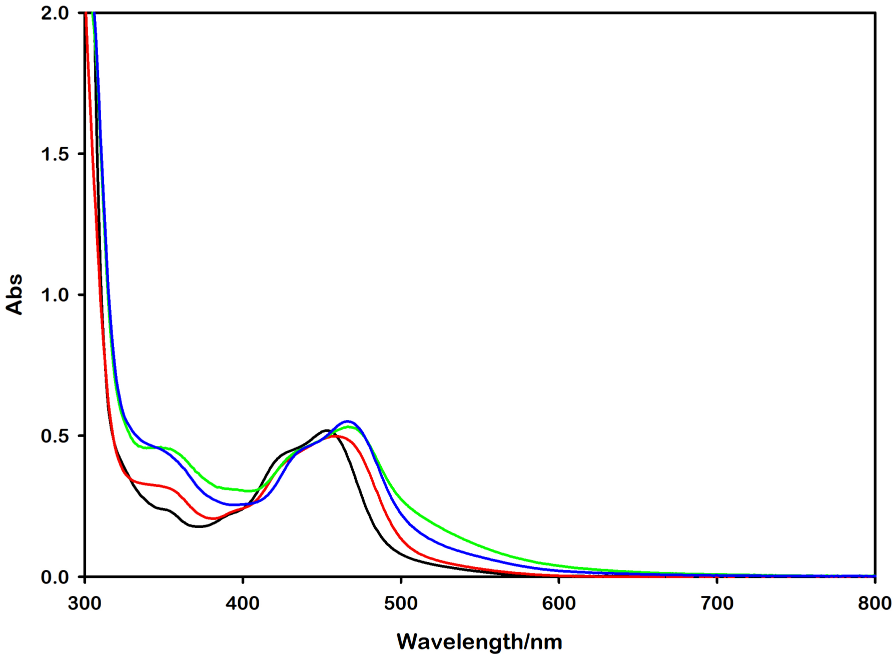

Figure S7. Absorption spectra of $[Ru(bpy)_3]^{2+}$ (black), $[Ru(bpy)_2(dcbpy)]$ (red), $[Ru(bpy)(dcbpy)_2]^{2-}$ (green), and $[Ru(dcbpy)_3]^{4-}$ (blue) reaction mixture for $MV^{\bullet+}$ photoaccumulation, each containing, in addition to Ru complex, 0.1 M TEOA and MV^{2+} to quench 50% of the excited states (pH = 10.50).

After degassing the samples, they were irradiated for 10 minutes under vigorous stirring at 460 nm using a high-power light-emitting diode (LED Engin LuxiGen™ LZ1-10B202-0000) operating at 50 mA and placed at a fixed distance of 10.0 cm from the cuvette quartz window.

The $MV^{•+}$ formation was then monitored by recording the absorption spectra of the solution at different irradiation times.

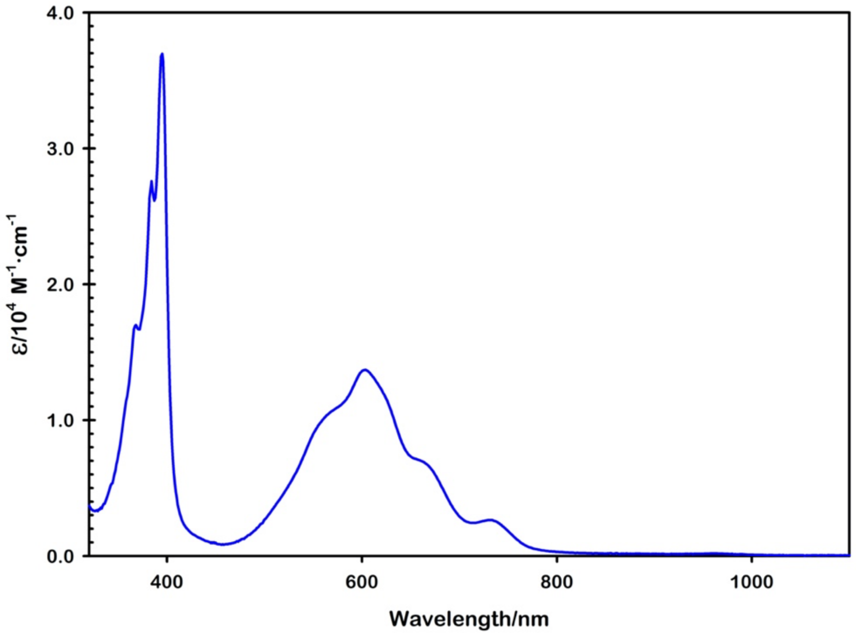

Figure S8. Molar absorption spectrum of $MV^{•+}$ in water.

[Ru(bpy)$_3$]$^{2+}$ – MV^{2+} irradiation

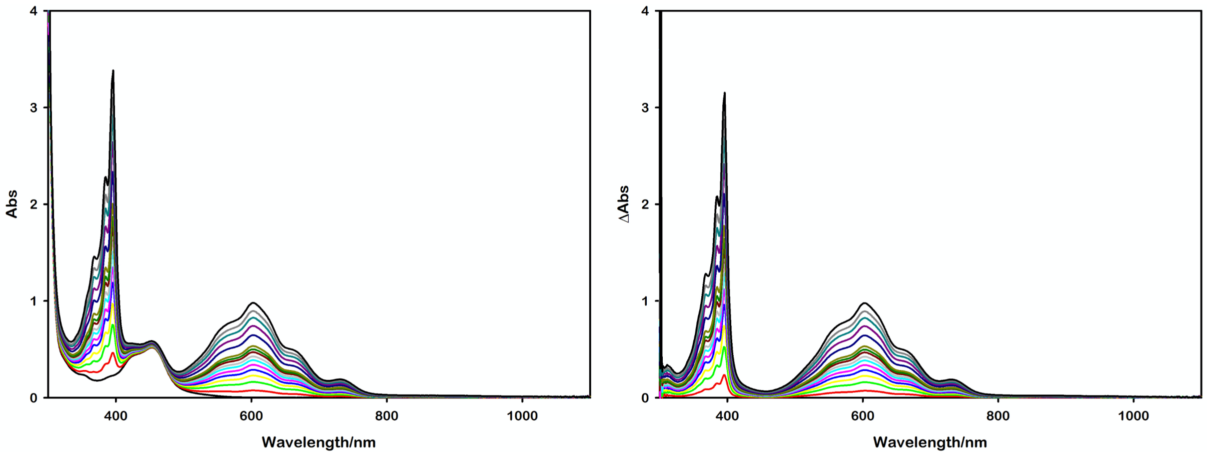

Figure S9. Absorption spectra of the [Ru(bpy)$_3$]$^{2+}$ – MV^{2+} and TEOA reaction mixture during irradiation (left panel). The right panel shows the corresponding arithmetic spectra.

[Ru(bpy)$_2$(dcbpy)] – MV^{2+} irradiation

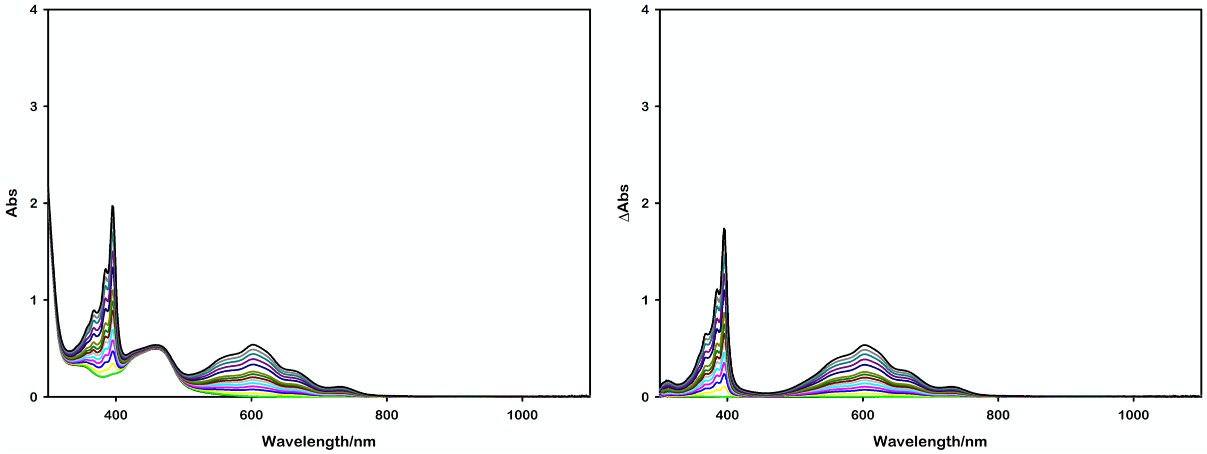

Figure S10. Absorption spectra of the [Ru(bpy)$_2$(dcbpy)] – MV^{2+} and TEOA reaction mixture during irradiation (left panel). The right panel shows the corresponding arithmetic spectra.

[Ru(bpy)(dcbpy)$_2$]$^{2-}$ – MV^{2+} irradiation

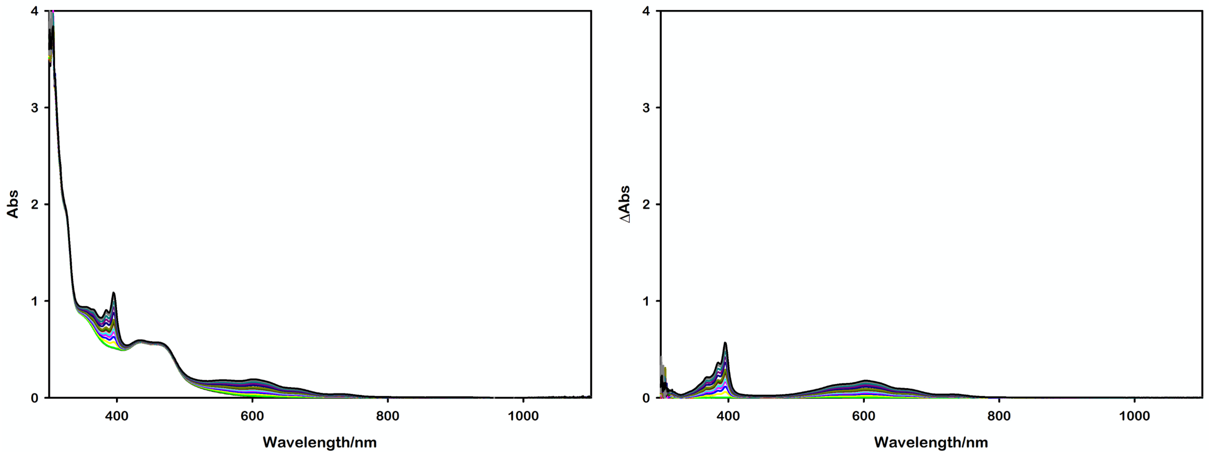

Figure S11. Absorption spectra of the [Ru(bpy)(dcbpy)$_2$]$^{2-}$ – MV^{2+} and TEOA reaction mixture during irradiation (left panel). The right panel shows the corresponding arithmetic spectra.

[Ru(dcbpy)$_3$]$^{4-}$ – MV^{2+} irradiation

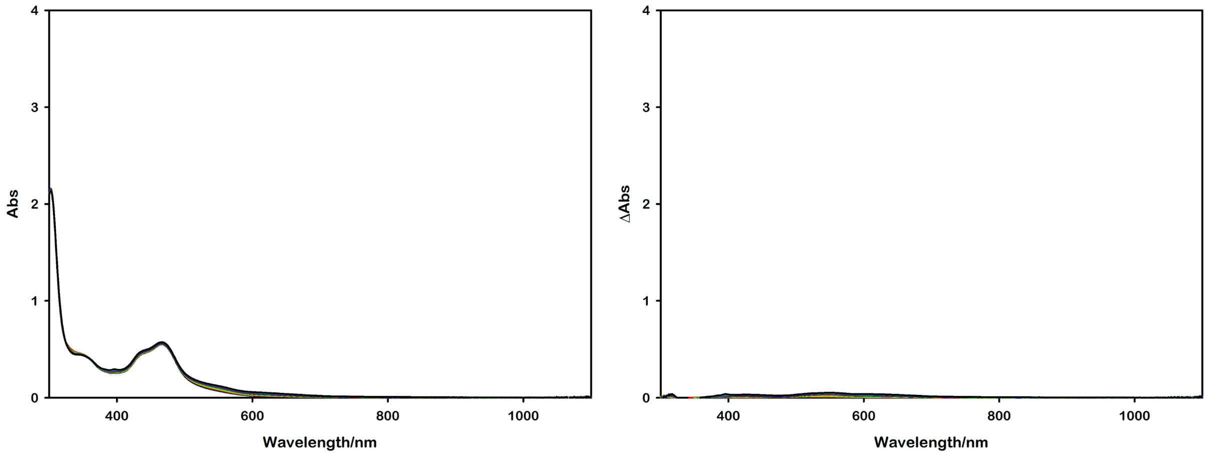

Figure S12. Absorption spectra of the [Ru(dcbpy)$_3$]$^{2-}$ – MV^{2+} and TEOA reaction mixture during irradiation (left panel). The right panel shows the corresponding arithmetic spectra.

CAGE ESCAPE QUANTUM YIELDS DETERMINATION

The number of photons absorbed by the samples was quantified via transient actinometry, using an air-equilibrated $[Ru(bpy)_3]^{2+}$ aqueous solution as a reference ($\Phi_{reference} = 1$),[S2,S3] ensuring the same 355 nm absorption as the samples. The actinometric procedure relies on monitoring the depletion of its ^{1}MCLT absorption band at 455 nm ($\Delta\varepsilon_{455\ nm} = -10\ 100\ M^{-1}\ cm^{-1}$).[S4]

The concentration of the transient $MV^{\bullet+}$ produced after the laser pulse was determined by recording its characteristic transient absorption signal at 605 nm ($\varepsilon_{605\ nm} = 13\ 700\ M^{-1}\ cm^{-1}$).[S5]

These concentrations were then compared as detailed in the Main Text.

$[Ru(bpy)_3]^{2+}$ – MV^{2+} transient actinometry

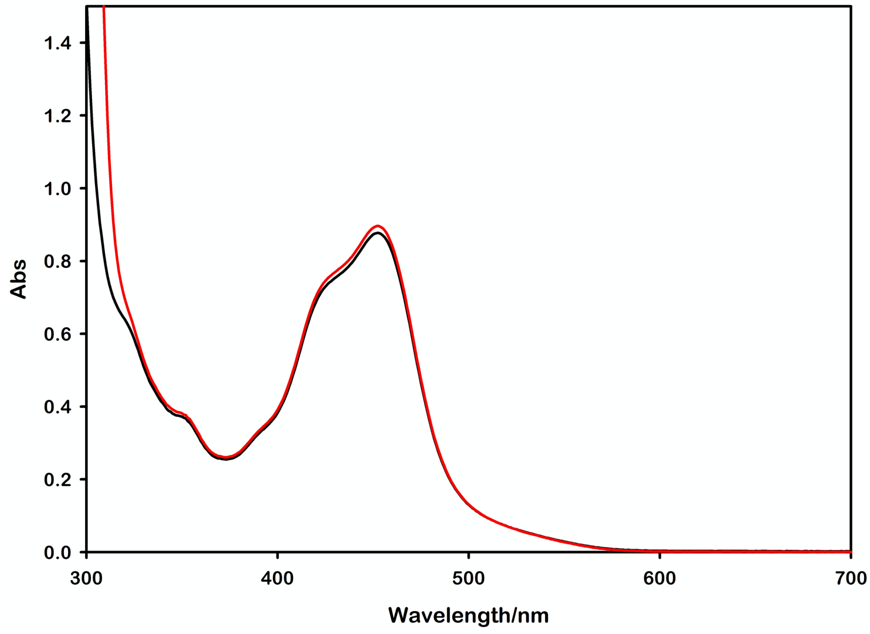

Figure S13. Absorption spectra of the $[Ru(bpy)_3]^{2+}$ solution (black) used as a standard for transient actinometry, and $[Ru(bpy)_3]^{2+}$ – MV^{2+} sample (red).

$[Ru(bpy)_2(dcbpy)]$ – MV^{2+} transient actinometry

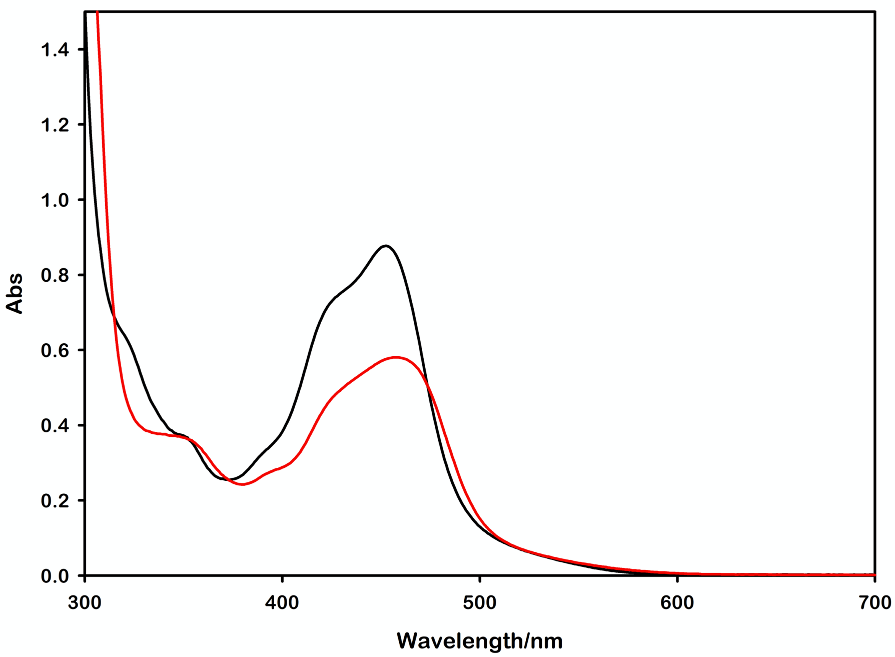

Figure S14. Absorption spectra of the $[Ru(bpy)_3]^{2+}$ solution (black) used as a standard for transient actinometry, and $[Ru(bpy)_2(dcbpy)]$ – MV^{2+} sample (red).

[Ru(bpy)(dcbpy)$_2$]$^{2-}$ – MV^{2+} transient actinometry

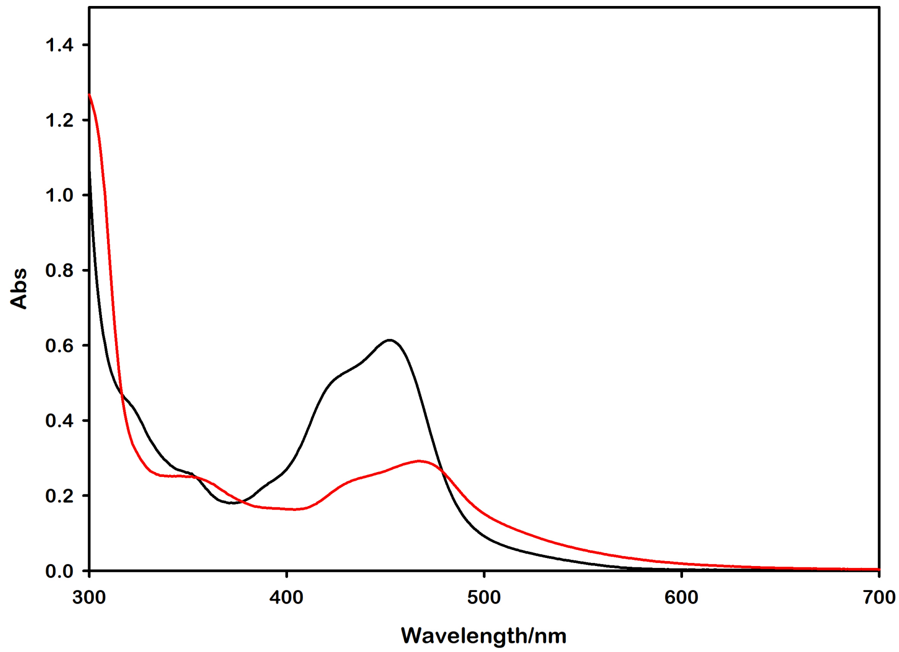

Figure S15. Absorption spectra of the [Ru(bpy)$_3$]$^{2+}$ solution (black) used as a standard for transient actinometry, and [Ru(bpy)(dcbpy)$_2$]$^{2-}$ – MV^{2+} sample (red).

[Ru(dcbpy)$_3$]$^{4-}$ – MV^{2+} transient actinometry

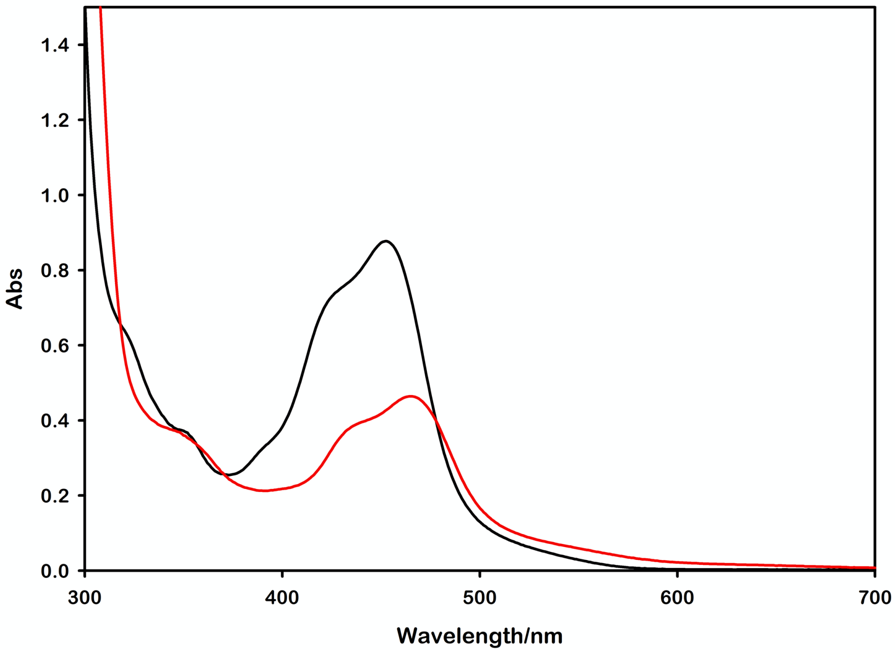

Figure S16. Absorption spectra of the [Ru(bpy)$_3$]$^{2+}$ solution (black) used as a standard for transient actinometry, and [Ru(dcbpy)$_3$]$^{4-}$ – MV^{2+} sample (red).

[Ru(bpy)(H$_2$dcbpy)$_2$]$^{2+}$ – MV^{2+} transient actinometry

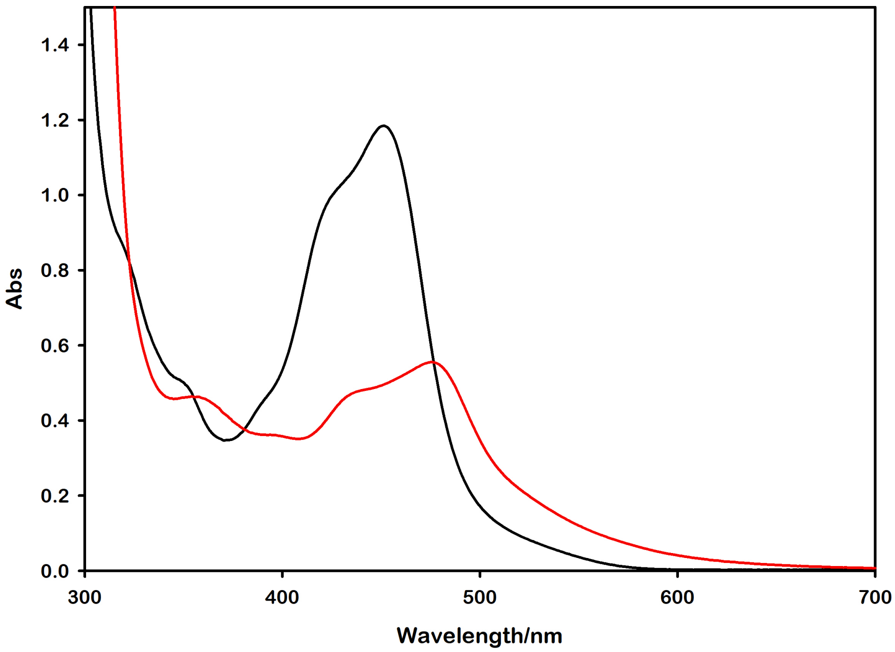

Figure S17. Absorption spectra of the $[Ru(bpy)_3]^{2+}$ solution (black) used as a standard for transient actinometry, and $[Ru(bpy)(H_2dcbpy)_2]^{2+}$ – MV^{2+} sample (red).

BACK-ELECTRON TRANSFER RATE CONSTANTS

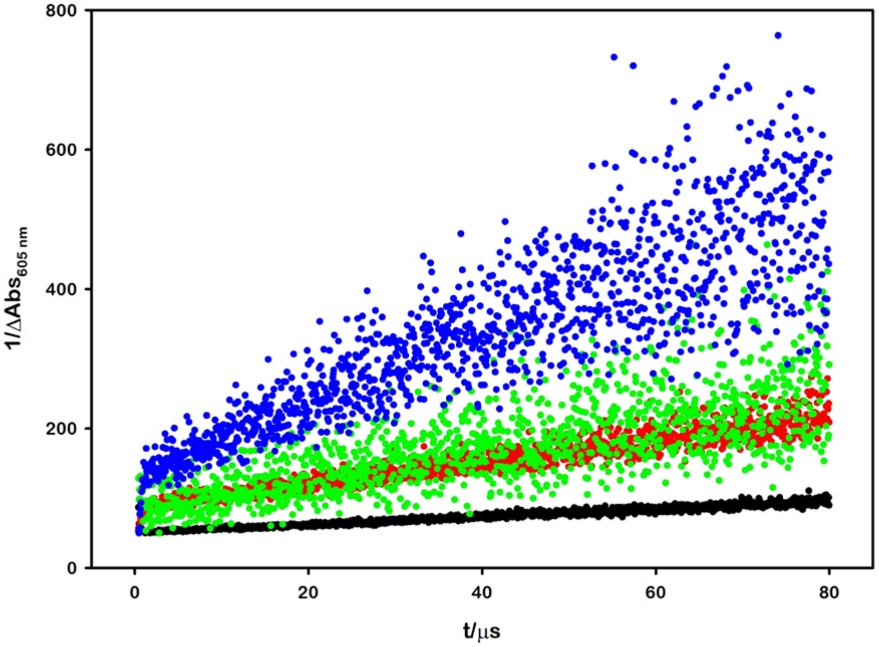

Figure S18. Second-order kinetic analysis of transient absorption at 605 nm for methyl viologen radical cation $MV^{•+}$ generated by laser flash photolysis (λ_{exc} = 355 nm) in 0.1 mM NaOH aqueous solution (pH = 10.00) by: $[Ru(bpy)_3]^{2+}$ (black dots), $[Ru(bpy)_2(dcbpy)]$ (red dots), $[Ru(bpy)(dcbpy)_2]^{2-}$ (green dots), and $[Ru(dcbpy)_3]^{4-}$ (blue dots).

REFERENCES

(S1) Park, H.; Bae, E.; Lee, J.-J.; Park, J.; Choi, W. Effect of the Anchoring Group in Ru–Bipyridyl Sensitizers on the Photoelectrochemical Behavior of Dye-Sensitized TiO 2 Electrodes: Carboxylate versus Phosphonate Linkages. *J Phys Chem B* **2006**, *110* (17), 8740–8749. https://doi.org/10.1021/jp060397e.

(S2) Ripak, A.; De Kreijger, S.; Elias, B.; Troian-Gautier, L. A Protocol for Determining Cage-Escape Yields Using Nanosecond Transient Absorption Spectroscopy. *STAR Protoc* **2023**, *4* (2), 102312. https://doi.org/10.1016/J.XPRO.2023.102312.

(S3) Wang, C.; Li, H.; Bürgin, T. H.; Wenger, O. S. Cage Escape Governs Photoredox Reaction Rates and Quantum Yields. *Nature Chemistry 2024 16:7* **2024**, *16* (7), 1151–1159. https://doi.org/10.1038/s41557-024-01482-4.

(S4) Neumann, S.; Kerzig, C.; Wenger, O. S. Quantitative Insights into Charge-Separated States from One- and Two-Pulse Laser Experiments Relevant for Artificial Photosynthesis. *Chem Sci* **2019**, *10* (21), 5624–5633. https://doi.org/10.1039/C9SC01381D.

(S5) Watanabe, T.; Honda, K. Measurement of the Extinction Coefficient of the Methyl Viologen Cation Radical and the Efficiency of Its Formation by Semiconductor Photocatalysis. *J Phys Chem* **1982**, *86* (14), 2617–2619. https://doi.org/10.1021/j100211a014.
